# Clinical efficacy of comprehensive traditional Chinese medicine in adjuvant therapy for stage Ib-IIIa resected non-small cell lung cancer: a multi-center, randomized, double-blind, placebo-controlled trial

**DOI:** 10.3389/fphar.2026.1776888

**Published:** 2026-04-09

**Authors:** Liping Shen, Yi Jiang, Xiaofeng Yang, Yuqing Cai, Ruoyan Qin, Xiaoli Xin, Juhua Yin, Xiangyan Bi, Peng Zhang, Aiqin Gu, Lei Jiang, Hecheng Li, ChunJie Li, Lingshuang Liu

**Affiliations:** 1 Department of Oncology, Longhua Hospital, Shanghai University of Traditional Chinese Medicine, Shanghai, China; 2 Department of Respiratory Medicine, Shanghai Chest Hospital, Shanghai, China; 3 Department of Thoracic Surgery, Ruijin Hospital, Shanghai Jiaotong University, Shanghai, China; 4 Department of Thoracic Surgery, Shanghai Pulmonary Hospital, Tongji University, Shanghai, China; 5 Hematology and Medical Oncology, Ohio State University Wexner Medical Center/The James Cancer Hospital, Columbus, OH, United States

**Keywords:** adjuvant chemotherapy, comprehensive traditional Chinese medicine, disease-free survival, radical resected non-small-cell lung cancer, randomized controlled trial

## Abstract

**Background:**

Recurrence and metastasis after radical resection for non-small cell lung cancer (NSCLC) remain a significant cause of treatment failure. Comprehensive Traditional Chinese Medicine (TCM) has emerged as a vital therapeutic approach in China. This study evaluated the efficacy and safety of TCM treatment with platinum-based adjuvant chemotherapy for patients of stage IB-IIIA resected NSCLC.

**Methods:**

A randomised controlled trial was conducted at four centres in Shanghai, China. A total of 286 participants with completely resected (R0) stage IB-IIIA NSCLC were randomly divided in a 1:1 ratio to a TCM treatment group (n = 143) and a control group (n = 143). Which received adjuvant chemotherapy plus TCM granules or placebo granules, for four cycles. And then, followed by four cycles of Huachansu injections. The received in addition to the adjuvant chemotherapy, followed by placebo injections.

**Results:**

ITT analysis was concluded with 286 patients. 2-year disease-free survival (DFS) rate (95% Confidence Interval [CI]) was 65.70% (57.86, 73.54) in the TCM treatment group and was 55.4% (47.17, 63.63) in the control group, the difference was not statistically significant, but a trend was observed (p = 0.08). A significantly prolonged DFS in the TCM group was revealed in contrast to the control group (37.8 months vs. 31.6 months, Hazard Ratio [HR], 0.73; 95% CI 0.579, 0.995; p = 0.045). TCM granules significantly improved lung cancer-related symptoms including fatigue, pain, dyspnea, insomnia, cough, chest pain and diarrhea, and promote physical function, emotional function and overall health. No unexpected treatment-related adverse events were observed in both groups.

**Conclusion:**

Comprehensive Traditional Chinese Medicine treatment combined with chemotherapy-based adjuvant therapy have a positive impact on DFS and improved quality of life in NSCLC patients. These findings suggested that TCM can be used as one of the adjuvant therapies following unselected radical resection in Chinese patients with stage IB-IIIA NSCLC.

**Clinical Trial Registration:**

https://www.chictr.org.cn/, identifier ChiCTR-IPR-16009062.

## 1 Introduction

Lung cancer is the malignant tumor with the highest morbidity and mortality. Non-small cell lung cancer (NSCLC) accounts for around 76% of lung cancers overall (Howlader et al., 2020). Surgical resection remains the primary treatment for stages I–III NSCLC. However, even after complete resection, the overall survival rate for patients with early-stage NSCLC remains low due to high rates of disease recurrence. Adjuvant standard cisplatin doublet chemotherapy decreases the risk of recurrence or death (Kris et al., 2017) and only improves the five-year survival rate of these patients by 5% (NSCLC Meta-analysis Collaborative Group, 2014). The Overall Survival (OS) benefit of adjuvant chemotherapy does not include patients with stage IA NSCLC (HR = 1.40) (Pignon et al., 2008). Therefore, adjuvant chemotherapy is not recommended for patients with stage IA NSCLC. Postoperative adjuvant therapy focuses primarily on patients with stage Ib-IIIa disease. Unfortunately, adjuvant chemotherapy plus bevacizumab appears to fail to improve overall survival due to toxicity (Wakelee, 2017).

Patients with advanced NSCLC and epidermal growth factor receptor (EGFR) mutations benefit clinically from EGFR tyrosine kinase inhibitors (TKIs) due to their excellent response rates and minor side-effect profiles. The efficacy of EGFR-TKIs in patients with advanced disease has led to their investigation as an adjuvant treatment for resectable disease (Tam, 2020). Three phase III trials were conducted in EGFR-mutant NSCLC: CTONG1104 (Zhong et al., 2021), EVIDENCE (He et al., 2021) and ADAURA (Tsuboi et al., 2023). These trials showed that EGFR-TKIs improved DFS and OS compared with chemotherapy. In contrast, the BR19 and RADIANT trials (Kelly et al., 2015) failed to demonstrate statistically significant improvements in either OS or DFS for gefitinib and erlotinib compared with placebo in patients with EGFR-mutation-unselected NSCLC. For resected NSCLC patients with stage IIIa, two-year disease-free survival was 81.4% (95% CI, 69.6–93.1) in the erlotinib group and 44.6% (95% CI, 26.9–62.4) in the chemotherapy group (relative risk 1.823 [95% CI, 1.194–2.784; p = 0.0054]) (Yue et al., 2018). Immune checkpoint inhibitors (ICIs) have proven their efficacy in advanced-stage NSCLC. Until now, neoadjuvant therapy has been associated with few side effects, does not delay surgery and induces a major pathological response in 45% of resected tumours (Forde et al., 2018; Eichhorn et al., 2021). In adjuvant treatment, atezolizumab improved DFS compared with best supportive care in patients with resected stage II–IIIA NSCLC followed by chemotherapy (HR, 0.79; 95% CI, 0.64–0.96; p = 0.020), especially in the subgroup whose tumour proportion score (TPS) expressed Programmed cell death ligand 1(PD-L1) ≥1% (HR, 0.66; 95% CI, 0.50–0.88; p = 0.0039) (Felip et al., 2021). Pembrolizumab has also been shown to significantly improve DFS compared with placebo in PD-L1-unselected stage IB-IIIA NSCLC (HR, 0.76; 95% CI, 0.63–0.91; p = 0.0014) (O’Brien et al., 2022).

For patients with stage IB–IIIA NSCLC after surgical resection, targeted drugs and immune checkpoint inhibitors (ICIs) have delayed recurrence and metastasis in some people not all. How can NSCLC patients who did not carry positive targets after surgery further prevent postoperative recurrence and metastasis? On the other hand, whether moving these targeted drugs and immunotherapies to adjuvant therapy can translate into a survival benefit remains inconclusive. Further study of the treatment model is needed.

Traditional Chinese medicine is widely used by Chinese patients with NSCLC, from tumour prevention to lung cancer surgery resection and even advanced NSCLC. Many studies have confirmed that TCM treatment can improve quality of life (QOL), reduce drug side effects, strengthen the efficacy of drugs, and prolong overall survival (Xi et al., 2025). In a previous study, adjuvant chemotherapy plus oral TCM improve QOL and performance status (Liu et al., 2023) and only prevented recurrence in stage IB resected NSCLC (Wang et al., 2020). Oral Chinese medicine has limited efficacy in preventing postoperative recurrence and metastasis of stage Ib-IIIa lung cancer. Therefore, Further clinical evidence is needed to confirm that traditional Chinese medicine can prolong disease-free survival (the most critical outcome indicator after lung cancer surgery) in the entire NSCLC postoperative population. Thus, in our clinical design, we extended the treatment duration of traditional Chinese medicine granules by 4 months and added the traditional Chinese medicine intravenous preparation, Huachansu (HCS). HCS is a water-soluble preparation made from the dried skin of the toad *Bufo gargarizans* or Bufo nigricollis (Meng et al., 2012), had been approved by the China Food and Drug Administration (CFDA) for the treatment of liver cancer, lung cancer, pancreatic cancer, and colorectal cancer (Yang et al., 2024). Based on these backgrounds, our research group has conducted a prospective, multicentre, randomised, double-blind, controlled study to evaluate the efficacy and safety of TCM in patients with completely resected stage IB–IIIA NSCLC.

## 2 Methods

### 2.1 Study design and study population

This multicentre, double-blind, randomised clinical trial recruited patients from four hospitals including Longhua Hospital, Shanghai University of Traditional Chinese Medicine, Shanghai Chest Hospital, Ruijin Hospital, Shanghai Jiaotong University, Shanghai Pulmonary Hospital, Tongji University in Shanghai between 1 November 2016 and 31 December 2018. The trial protocol has also been published (Jiang et al., 2019). No changes were made to the methods after the commencement of the trial.

The primary endpoint was the two-year DFS rate, defined as the percentage of patients without disease recurrence or death within 2 years after randomisation. Key secondary endpoints included DFS, quality of life, and regimen safety. DFS was measured from the date of randomisation until the first recurrence or death from any cause. Computed tomography (CT) scans of the chest were performed every 3 months to evaluate recurrence, and then every 6 months until death or the analysis cutoff date. CT or magnetic resonance imaging of the upper abdomen and brain, as well as bone emission CT, were performed if clinically indicated. Quality of life was assessed using the European Organisation for Research and Treatment of Cancer (EORTC) Quality of Life Questionnaire Core 30 (QLQ-C30) and the associated EORTC Quality of Life Lung Cancer Specific Module (QLQ-LC13) at baseline and at the end of the second treatment cycle. Adverse events were monitored for using the National Cancer Institute’s Common Terminology Criteria for Adverse Events v3.0 each week during chemotherapy, and before and after each TCM treatment cycle.

To ensure the reliability and operability of the results, this study used paired randomization. Patients were stratified according to disease stage (IB, II, or IIIA) and TCM syndrome differentiation (Qi deficiency, Yin deficiency, or both), were randomly assigned in a 1:1 ratio to receive TCM granules orally every day or placebo for 6 months. Random numbers will be generated automatically using a computer according to pre-configured stratified factors (pathological stage and histological subtype). The Randomization Program was designed by statistician Huang Pin-xian at Shanghai University of TCM. Central randomisation was performed via telephone.

In this study, participants, investigators, outcome assessors and statisticians will be unaware of the allocated treatment. The same research product was used in terms of appearance, packaging and labelling to ensure blinding.

The study had been approved by the ethics committee of Longhua Hospital, affiliated with Shanghai University of TCM, Shanghai, PR China (ethical approval 2016LCSY039). Furthermore, the trial was registered by the Chinese Clinical Trials with the following code: ChiCTR-IPR-16009062 (https://www.chictr.org.cn/).

### 2.2 Sample size calculation

Referring to the results of the meta-analysis of 4,584 patients with non-small cell lung cancer (NSCLC) who received platinum-containing adjuvant chemotherapy after complete resection, the two-year DFS rate was 60% (Pignon et al., 2008). Based on this result and the traditional Chinese medicine treatment used in our study, it was estimated that the two-year DFS rate could be increased to 75%. With a test level of α = 0.05 and a power of 1-β = 0.8, the calculated sample size was 260 cases. With a dropout rate of up to 10%, a total of 286 patients were planned.

### 2.3 Inclusion criteria

Patients were eligible for inclusion in the study if they meet the following criteria: (1) Study participants were individuals aged 18–75 years with histologically confirmed NSCLC; (2) patients completely resected stage IB-IIIA disease within 6 weeks; (3) Eastern Cooperative Oncology Group (ECOG) Performance Status (PS) score was 0–2; (4) TCM syndromes including qi deficiency, yin deficiency or qi and yin deficiency; (5) voluntary participation in the clinical study, normal haematological function with a total neutrophil count of >1.5 × 10^9^/L and a platelet count of >80 × 10^9^/L, normal liver and kidney function and signed informed consent.

### 2.4 Exclusion criteria

Patients with the following conditions will be excluded: (1) Presence of other primary malignancies; (2) Patients with severe concomitant diseases of the heart, liver, kidneys or haematopoietic system; (3) pregnant or breastfeeding patients; (4) allergy to the study medication; (5) Patients with a history of uncontrollable psychiatric disorders; (6) All patients provided informed consent prior to participation; (7) Any severe, unstable, or concurrent medical condition that could interfere with the study protocol; (8) Patients who developed recurrence or metastasis within 4 months after surgery.

### 2.5 Therapeutic regimen

Patients will be administered platinum-based chemotherapy according to one of the following regimens, for a total of four cycles of 3 weeks each, as per the National Comprehensive Cancer Network guidelines: (1) GP regimen: gemcitabine (1250 mg/m^2^) on days 1 and 8, and cisplatin (75 mg/m^2^) on day 1; (2) DP regimen: docetaxel 75 mg/m^2^ on day 1 and cisplatin 75 mg/m^2^ on day 1; (3) PP regimen: pemetrexed 500 mg/m^2^ on day 1 (for non-squamous NSCLC) and cisplatin 75 mg/m^2^ on day 1; (4) TC regimen: paclitaxel 135 mg/m^2^ on day 1 and carboplatin area under the curve 5 on day 1. Patients who cannot tolerate cisplatin will be given carboplatin with an area under the curve of 5 on day 1. All chemotherapeutic agents will be administered intravenously in both groups. Adjuvant radiation therapy is not recommended for routine use (Kris et al., 2017). Postoperative radiotherapy for patients with stage IIIA-N2 disease is at the discretion of the researchers at the participating centres.

According to the “Guidelines of Diagnosis and Therapy in Oncology with Traditional Chinese Medicine”, patients would receive a huachansu injection (20 mL added to a 500 mL 0.9% sodium chloride solution per day for days 1–10) every 28 days for four cycles after four cycles of chemotherapy. The Huachansu injection comes from a single batch (170301-2) manufactured by Anhui Jinchan Biochemistry Shareholders. Saline would be used as a placebo and administered on the same schedule as the Huachansu injection.

During adjuvant chemotherapy and huachansu injection treatment, all patients took TCM granules orally every day. Patients were categorised according to TCM syndrome differentiation as having either Qi deficiency, Yin deficiency, or both. Jia-xiang Liu (Longhua Hospital, affiliated with Shanghai University of Traditional Chinese Medicine) prescribed Chinese herbal medicine for the patients. This was classified into four categories according to its function: recipes to benefit Qi, Yin, both Qi and Yin, and to detoxify and eliminate lumps and tumours. The herbal and placebo granules were water-soluble and manufactured in accordance with Good Manufacturing Practice standards at a facility in China (Jiangyin Tian Jiang Pharmaceutical Co.). The Syndrome Differentiation Criteria and the method of taking TCM were detailed in the published protocol.

The drug composition and dosages of the four traditional Chinese medicine prescriptions in this study are as follows: (1) The ingredients in the qi-boosting recipe included Radix Astragali (Huangqi) 15 g, Rhizoma Atractylodis Macrocephalae (Baizhu) 12 g, Poria (Fuling) 15 g, Herba Epimedii (Yinyanghuo) 15 g, and Semen Trigonellae (Huluba) 15 g. (2) The ingredients in the benefiting yin recipe include Radix Adenophorae (Nanshashen) 30 g, Radix Glehniae (Beishashen) 30 g, Radix Asparagi (Tiandong) 15 g, Radix Ophiopogonis (Maidong) 15 g, Bulbus Lilii (Baihe) 12 g, Fructus Ligustri Lucidi (Nvzhenzi) 12 g, and Fructus Corni (Shanyurou) 12 g. (3) The ingredients in the benefiting qi and yin recipe included Radix Astragali (Huangqi) 15 g, Radix Glehniae (Beishashen) 30 g, Radix Asparagi (Tiandong) 15 g, Radix Ophiopogonis (Maidong) 15 g, Fructus Ligustri Lucidi (Nvzhenzi) 12 g, Fructus Corni (Shanyurou) 12 g, Semen Trigonellae (Huluba) 12 g, and Herba Epimedii (Yinyanghuo) 15 g. (4) The ingredients in the detoxication and resolving masses recipe include Selaginella doederleinii Hieron (Shishangbai) 15 g, Salvia chinensis Benth (Shijianchuan) 15 g, Paris polyphylla (Qiyeyizhihua) 7.5 g, Pseudobulbus Cremastrae Seu Pleiones (Shancigu) 7.5 g, Herba Euphorbia-helioscopia (Zeqi) 7.5 g, Spica Prunellae (Xiakucao) 7.5 g, Rhizoma Arisaematis (Tiannanxing) 7.5 g, Rhizoma Amorphophalli (Sheliugu) 15 g, and Fructus Jujubae (Dazao) 6 g. The placebo granules contained no Chinese herbal medicine, but matched the Chinese herbal granules in terms of weight, colour, smell, taste and packaging. The four prescriptions, based on the aforementioned drugs and dosages, were processed into corresponding granules. Each package of benefiting qi recipe contained 4.8 g of water-soluble herbal granules, Each package of benefiting yin recipe contained 12 g of water-soluble herbal granules, Each package of benefiting qi and yin recipe contained 10 g of water-soluble herbal granules, and Each package of detoxication and resolving masses recipe contained 4 g of water-soluble herbal granules. Patients took the granules twice daily, orally four packages each time, including two packages of detoxication and resolving masses granules and two packages of benefiting qi granules or benefiting yin granules or benefiting qi and yin granules. The four packages’ granules dissolved in 150 mL of warm water for drinking, to be taken 30 min after a meal in the morning and evening.

The trial conducted interim analyses at two time points: 1 year and 2 years after the start of the study. Common reasons for premature termination of trials in interim analysis: (1) Unacceptable adverse reactions or drug toxicity are discovered, posing a safety risk; (2) The enrollment rate is too slow, making it difficult to continue the trial; (3) The trial’s execution is so poor that the intended objectives cannot be achieved.

To detect adverse outcomes, physicians conducted weekly follow-up visits with patients undergoing chemotherapy. Four months after completing chemotherapy, patients underwent monthly follow-up visits.

### 2.6 Statistical methods

Intention-to-treat (ITT) analysis included all participants and the Per-Protocol (PP) analysis included 131 participants to TCM treatment group and 132 to control group. All statistical analyses were performed using SPSS 25.0. A two-sided P value of less than 0.05 was considered statistically significant. We then used the chi-square test to compare the two-year DFS rate between patients who received adjuvant chemotherapy plus TCM granules and those who received adjuvant chemotherapy plus placebo granules. DFS survival curves were estimated using the Kaplan-Meier method and compared between the two groups using the log-rank test. Hazard ratios and their respective 95% CIs were assessed using a stratified Cox proportional hazards model. Subgroup analyses were performed in subgroups by baseline age, sex, pathology, Lumph-node stage, and TCM syndrome. Changes in EORTC QLQ-C30 and EORTC QLQ-LC13 questionnaire scores from baseline to the end of two cycles were assessed using the Wilcoxon rank sum test. Safety was assessed in all eligible patients who received at least one dose of treatment. All statistical tests were carried out on the basis of a two-sided α of 0.05 and a 95% confidence interval. The statistical analysis was conducted by experts from the Department of Health Statistics at Shanghai University of TCM.

## 3 Results

### 3.1 Patients’ information

**FIGURE 1:**
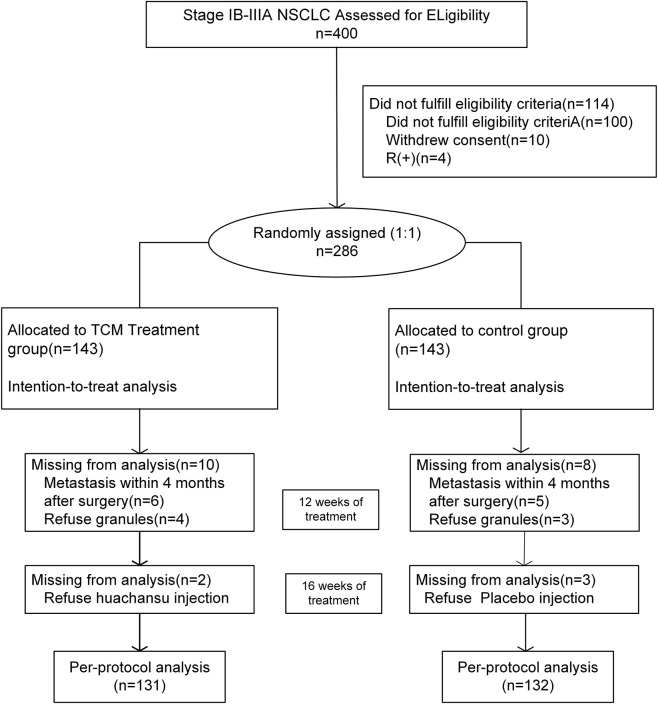
CONSORT-inclusion and exclusion flowchart.

400 patients were screened and 286 were enrolled and randomized from 1 November 2016, and 31 December 2018. 21 cases (12 cases in the treatment group and 11 cases in the control group) were excluded because of disease recurrence and metastasis occurred within four months after the operation, rufusing TCM granules, and injection treatment. 131 of 143 patients allocated treatment group and 132 of 143 patients allocated control group were included in the PP population analysis and safety population analysis (Figure 1). As the data cutoff of Aug. 31, 2025, 36 patients in the treatment group and 21 patients in the control group are still being followed up.

Baseline characteristics were well balanced between two groups (Table 1). About three-fifth of patients were male, and the median age of the patients was 60 years (IQR 51–66). Most patients had adenocarcinoma (77.86% in the treatment group and 75% in the control group), with stage IIIA disease (32.82% and 34.09%). In the patients with N2, 43.58% (17/39) in the treatment group and 37.78% (17/45) in the control group had radiotherapy.

**TABLE 1:** Baseline characteristics.

Items	Treatment group (n = 131)	Control group (n = 132)	Test value	*P* value
Sex				
Male	81 (61.83%)	79 (59.84%)	χ^2^ = 0.742	0.801
Female	50 (38.16%)	53 (40.15%)		
Age				
Median (range), years	61 (33–75)	60 (37–75)	χ^2^ = 0.002	0.965
≥65 years	44 (33.59%)	44 (33.33%)		
<65 years	87 (66.41%)	88 (66.66%)		
Pathological typing				
Adenocarcinoma	102 (77.86%)	99 (75.00%)	χ^2^ = 5.046	0.135
Squamous	24 (18.32%)	31 (23.48%)		
Other-NSCLC	5 (3.8%)	2 (1.51%)		
Disease stage				
IB	53 (40.46%)	49 (37.12%)	χ^2^ = 0.597	0.898
II	35 (26.72%)	38 (28.79%)		
IIIA	43 (32.82%)	45 (34.09%)		
T				
1	17 (12.98%)	22 (16.67%)	χ^2^ = 1.228	0.746
2	93 (70.99%)	93 (70.45%)		
3	19 (14.50%)	16 (12.12%)		
4	2 (1.53%)	1 (0.76%)		
N				
0	68 (51.91%)	69 (52.27%)	χ^2^ = 1.289	0.525
1	24 (18.32%)	18 (13.64%)		
2	39 (29.77%)	45 (34.09%)		
Chemotherapy				
PP/PC	101 (77.10%)	96 (72.73%)	χ^2^ = 1.766	0.638
TP/TC	5 (3.82%)	9 (6.82%)		
GP/GC	22 (16.79%)	22 (16.67%)		
DP/DC	3 (2.29%)	5 (3.78%)		
Chemotherapy cycle				
1	6 (4.58%)	5 (3.79%)	χ^2^ = 6.736	0.081
2	7 (5.34%)	1 (0.76%)		
3	0	2 (1.51%)		
4	118 (90.08%)	124 (93.94%)		
Radiotherapy				
Yes	17 (12.98%)	17 (12.88%)	χ^2^ = 0.001	0.981
None	114 (87.02%)	115 (87.12%)		
TCM syndrome differentiation				
Qi deficiency syndrome	64 (48.85%)	75 (56.82%)	χ^2^ = 1.734	0.420
Yin deficiency syndrome	8 (6.11%)	6 (4.54%)		
Qi and yin deficiency syndrome	59 (45.04%)	51 (38.64%)		

### 3.2 Effect of TCM treatment on two-year DFS rate and DFS

During the postoperative adjuvant therapy period, 90.08% patients in the TCM treatment group and 93.94% patients in the control group completed four cycles of chemotherapy (Table 1).

At the data cutoff, the median follow-up period was 90.23 months. A total of 98 (68.53%) patients in the TCM treatment group and 113 (79.02%) patients in the control group experienced disease recurrence or metastasis or death. Of the patients who experienced the endpoint event, one patient in the TCM treatment group and two patients in the control group died without disease progression or relapse. However, 18 patients were not available for follow-up of DFS.

**FIGURE 2:**
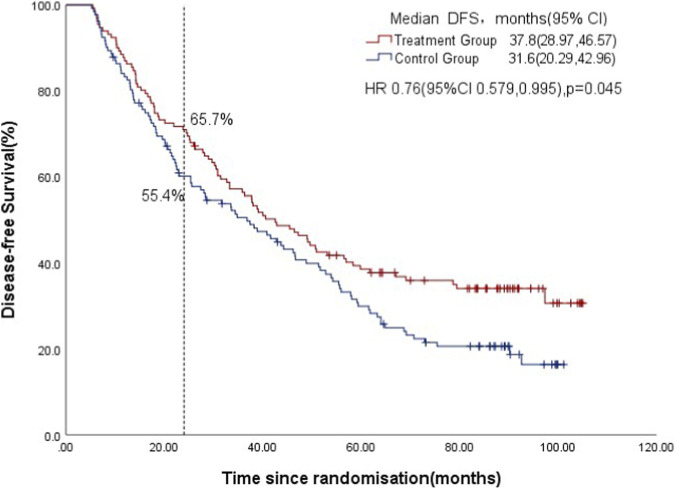
Kaplan-Meier estimates of disease-free survival between two group (ITT analysis, n = 286).

In ITT population analysis, a total of 286 patients randomly divided into two groups: 143 in the TCM treatment group and 143 in the control group. The two-year DFS rate (95% CI) was 65.70% (57.86, 73.54) in the treatment group, in comparison to 55.4% (47.17, 63.63) in the control group (p = 0.08); And disease-free survival among all patients was 37.8 months (95% CI 28.97, 46.57) in the TCM treatment group and 31.6 months (95% CI 20.29, 42.96) in the control group (HR, 0.73 [95% CI, 0.579, 0.995]; p = 0.045; Figure 2).

**FIGURE 3:**
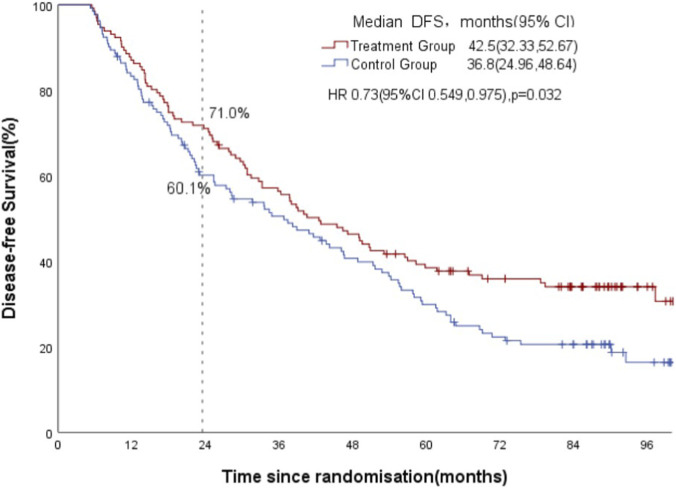
Kaplan-Meier estimates of disease-free survival between two group (PP analysis, n = 263).

**FIGURE 4:**
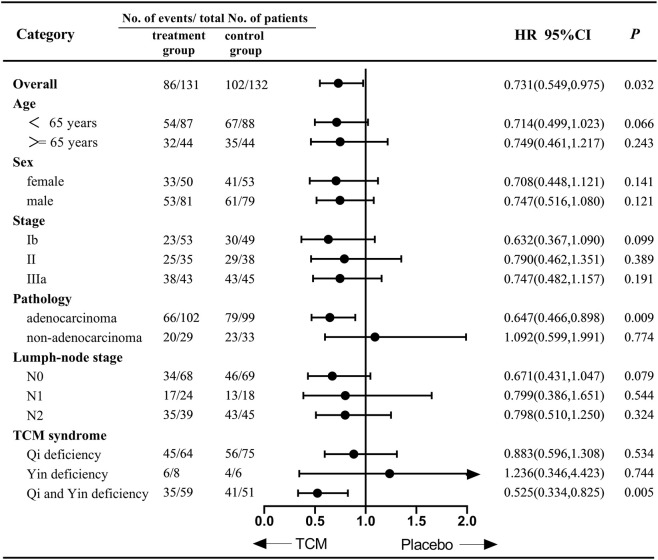
Subgroup analysis of disease -free survival according to baseline characteristics.

In PP population analysis, the two-year DFS rate and DFS were compared between 131 patients in the treatment group and 132 patients in the control group. The primary endpoint of the two-year DFS rate (95% CI) was 71.00% (63.14, 78.84) in the treatment group, in comparison to 60.10% (51.67, 68.53) in the control group. The difference in the two-year DFS rate between the two groups was not statistically significant (p = 0.071). The treatment group demonstrated a superior DFS in comparison to the control group (HR, 0.73 [95% CI, 0.549, 0.975]; p = 0.032; Figure 3). The median DFS was found to be 42.5 months (95% CI 32.33, 52.67) in contrast to 36.8 months (95% CI 24.96, 48.64). Furthermore, subgroup analysis indicates that patients diagnosed with adenocarcinoma and both qi and yin deficiency syndromes may achieve improved DFS following traditional Chinese medicine treatment (Figure 4).

### 3.3 Effect on quality of life

The QLQ-C30, a 30-item questionnaire, included five functional scales (physical, role, cognitive, emotional, and social), three symptom items (fatigue, pain, nausea, and vomiting), and global health and QOL scales. The QLQ-LC13 is a 13-item scale designed to evaluate symptoms associated with lung cancer, including cough, hemoptysis, and dyspnoea, as well as treatment-related adverse effects such as sore mouth or tongue, dysphagia, hair loss, tingling in the hands and feet, pain, and the need for analgesics. The measurement of outcomes was conducted at the baseline stage, and then at the conclusion of each two-cycle period.

#### 3.3.1 Within group comparison of the QOL of in two groups before and after treatment

The Nonparametric tests were used to count the changes of the EORTC QLQ-C30 scale and QLQ-LC13 scale in the two groups between before and after 2-cycle treatment. A dominant positive rank sum indicates an increase in score after treatment, while a dominant negative rank sum indicates a decrease in score after treatment. The scores of the symptom items in the C30 scale and each LC13 item decreased, with negative ranks predominating, suggesting symptom relief after treatment.

The findings of the EORTC QLQ-C30 scale demonstrated that the physical function, emotional function and global health of the treatment group following treatment exhibited a positive rank sum that was predominant (p < 0.01). Conversely, the negative rank sum of fatigue, pain, shortness of breath, insomnia and diarrhoea was predominant. The differences were statistically significant (p < 0.01, p < 0.01, p < 0.05, p < 0.01, p < 0.05). The positive rank sum of physical function in the control group exhibited dominance post-treatment, with a statistically significant difference (P < 0.01). Concurrently, the negative rank sum of dyspnea and insomnia manifested dominance, yielding a statistically significant difference (p < 0.01). It was hypothesised that the combination of adjuvant chemotherapy and TCM granules would enhance physical function, emotional wellbeing and overall health, while concomitantly reducing symptoms of fatigue, pain, dyspnoea, insomnia and diarrhoea (Table 2).

**TABLE 2:** Within-group Comparison of the QOL in the two groups before and after 2-cycle treatment.

Items	Treatment group (n = 131)				Control group (n = 132)			
	Positive rank sum	Negative rank sum	Z	*P*	Positive rank sum	Negative rank sum	Z	*P*
EORTC QLQ-C30								
Physical functioning	3024.5	630.5	−5.256	0.000^a^	3926.0	634.0	−6.147	0.000^a^
Role functioning	1061.5	826.5	−0.840	0.401	1104.5	606.5	−1.948	0.051
Emotional functioning	2050.5	950.0	−6.413	0.000^a^	571.0	332.0	−1.502	0.133
Cognitive functioning	626.0	1265.0	−2.343	0.019^b^	444.0	781.0	−1.758	0.079
Social functioning	268.5	511.5	−1.173	0.087	402.5	500.5	−0.624	0.533
Global health	2366.0	484.0	−4.991	0.000^a^	1018	1757	−1.733	0.045
Fatigue	504.0	2197.0	−4.685	0.000^a^	1182.5	1095.5	−0.273	0.785
Nausea	24	12	−0.905	0.366	43	12	−1.645	0.100
Pain	224.5	678.5	−3.040	0.002^a^	245.5	420.5	−1.470	0.142
Dyspnea	260.5	600.5	−2.048	0.016^b^	460.0	1251.0	−3.284	0.001^a^
Insomnia	81.0	739.0	−4.585	0.000^a^	213.5	689.5	−3.118	0.002^a^
Appetite loss	132.0	144.0	−0.196	0.845	132.0	246.0	−1.454	0.146
Constipation	39.5	38.5	−0.042	0.967	99.5	53.5	−1.166	0.243
Diarrhea	15.0	105.0	−2.626	0.009^a^	97.0	56.0	−1.507	0.291
Financial problems	259.5	236.5	−0.232	0.817	358.0	203.0	−1.408	0.159
EORTC QLQ-LC13								
Short of breath	106.0	5889.0	−8.758	0.000^a^	46.5	5413.5	−8.712	0.000^a^
Cough	279.0	2349.0	−6.296	0.000^a^	311.5	769.5	−2.677	0.007^a^
Hemoptysis	6.0	4.0	−0.378	0.705	0.00	3.00	−1.414	0.157
Sore mouth or tongue	44.0	22.0	−1.069	0.285	17.5	37.5	−1.040	0.298
Trouble in swallowing	4.0	6.0	−0.378	0.705	15.0	30.0	−1.000	0.317
Tingling hands or feet	14.0	14.0	−0.000	1.000	14.0	14.0	0.000	1.000
Hair loss	16.0	20.0	−0.302	0.763	0.0	3.0	−1.414	0.157
Pain in chest	117.0	744.0	−4.519	0.000^a^	120.0	376.0	−2.796	0.005^a^
Pain in arm or shoulder	63.0	90.0	−0.667	0.505	100.0	200.0	−1.521	0.128
Pain in other parts of body	16.5	49.5	−1.604	0.109	12.5	32.5	−1.208	0.227

QLQ-C30, Quality of Life Questionnaire Core 30; QLQ-LC13, Quality of Life Questionnaire Lung Cancer-Specific Module 13.

^a^ Compared with baseline, the difference of treatment group or control group in2-cycle treatment was statistically significant (*P* < 0.01).

^b^ Compared with baseline, the difference of treatment group or control group in2-cycle treatment was statistically significant (*P* < 0.05).

The results of QLQ-LC13 scale showed: the treatment group after 2-cycle treatment, negative rank sum in short of breath, cough, trouble in swallowing, hair loss, pain in chest, pain in arm or shoulder, pain in other parts was dominant. While the positive rank sum in hemoptysis, Sore mouth or tongue was dominant. However, only the differences in shortness of breath, cough and pain in chest were statistically significant (p < 0.01). In the control group after treatment, negative rank sum in short of breath, cough, hemoptysis, Sore mouth or tongue, trouble in swallowing, hair loss, pain in chest, pain in arm or shoulder, pain in other parts was dominant, only the differences in shortness of breath, cough and pain in chest were statistically significant (p < 0.01) (Table 2).

#### 3.3.2 Comparison between groups of the QOL with baseline and after two-cycles’ treatment

The results of EORTC QLQ-C30 scale: In baseline, there was no significant difference in the scores of various fields between the two groups (p > 0.05). After two-cycles’ treatment, the sum rank of emotional function and Global health in the treatment group was higher than that in the control group, the differences were statistically significant (p < 0.05, p < 0.01). The sum rank of fatigue and diarrhea in the treatment group was lower than that in the control group, the differences were statistically significant (p < 0.05). It is suggested again that the adjuvant chemotherapy combined with TCM can improve the emotional function and global health, reduce the symptoms of fatigue and diarrhea (Table 3).

**TABLE 3:** Comparison between groups of the QOL before and after 2-cycle treatment.

Items	Baseline				After 2-Cycles’ Treatment			
	Treatment group		Control group		Treatment group		Control group	
	Sum rank	Average rank	Sum rank	Average rank	Sum rank	Average rank	Sum rank	Average rank
EORTC QLQ-C30								
Physical functioning	16092.5	128.7	16038.5	125.3	16240.5	129.9	15890.5	124.1
Role functioning	15690.5	125.5	16440.5	128.4	15443.0	123.5	16688.0	130.4
Emotional functioning	14288.0	114.3	17843.0	139.4	16819.5	134.5	15311.5	119.6^a^
Cognitive functioning	16097.5	128.8	16033.5	125.3	15753.0	126.0	166378.0	127.9
Social functioning	16058.0	128.5	16073.0	125.6	15715.0	125.7	16416.0	128.2
Global health	14972.0	119.8	17159.0	134.05	17848.0	142.7	14283.0	111.6^b^
Fatigue	16649.5	133.2	15481.5	120.9	14661.0	117.2	17470.0	136.5^a^
Nausea	16012.0	128.1	16119.0	125.9	16007.0	128.1	16124.0	125.9
Pain	16050.5	128.4	16080.5	125.6	15294.5	122.3	16837.0	131.5
Dyspnea	16180.5	129.4	15950.5	124.6	16637.0	133.1	15494.0	121.1
Insomnia	15997.0	127.9	16134.0	126.5	15642.5	125.1	16488.5	128.8
Appetite loss	15510.5	124.1	16620.5	129.9	15789.0	126.3	16342.0	127.6
Constipation	16225.5	129.8	15905.5	124.3	15314.5	122.5	16816.5	131.8
Diarrhea	15825.0	126.6	16306.0	127.4	15695.5	125.5	16435.5	128.4^a^
Financial problems	15870.5	126.9	16260.5	127.0	15425 0.0	123.4	16706.0	130.5
EORTC QLQ-LC13								
Short of breath	15690.0	125.5	16441.0	128.45	16151.5	129.21	15979.5	124.8
Cough	16614.0	132.9	15517.0	121.23	13982.5	111.86	18148.5	141.8^b^
Hemoptysis	15942.0	127.5	16189.0	126.5	16067.0	128.5	16064.0	125.5
Sore mouth or tongue	15747.5	126.0	16383.5	128.0	16011.0	128.1	16120.0	125.9
Trouble in swallowing	15820.0	126.6	15800.5	123.4	15881.0	127.1	16250.0	126.9
Tingling hands or feet	15817.5	126.5	16313.5	127.4	15818.5	126.5	16312.5	127.4
Hair loss	15945.0	127.6	16186.0	126.4	16068.5	128.6	16062.5	125.5
Pain in chest	16580.0	132.6	15551.0	121.49	15435.0	123.5	16696.0	130.44
Pain in arm or shoulder	15702.5	125.6	16428.5	128.35	15730.0	125.8	16401.0	128.1
Pain in other parts of body	15944.0	127.6	16187.0	126.5	15818.5	126.5	16312.5	127.4

^a^ The difference between two groups was statistically significant (*P* < 0.05).

^b^ The difference between two groups was statistically significant (*P* < 0.01).

The results of EORTC QLQ-C13 scale: In baseline, there was no significant difference in the scores of various fields between the two groups (p > 0.05). After two-cycles’ treatment, the sum rank of cough in the treatment group was lower than that in the control group, and the difference in cough was statistically significant (p < 0.01). It was suggested that the adjuvant chemotherapy combined with TCM granules can reduce the symptoms of cough (Table 3).

### 3.4 Adverse effects

Most patients had completed four cycles adjuvant chemotherapy (Table 1). 13 in treatment group and eight in control group had only 1–3 cycles adjuvant chemotherapy. More patients required dose or chemotherapy drug adjustments as a result of AEs in the control group (n = 25; 18.93%) than in the treatment group (n = 15; 11.45%). Treatment-related adverse events were reported for 101 (77.10%) patients treated with TCM granule plus adjuvant chemotherapy and 102 (77.27%) patients treated with placebo plus adjuvant chemotherapy. 44 (16.73%) experienced grade 3 or greater toxicities with adjuvant therapy (19 [14.50%] patients in the treatment group vs. 24 [18.18%] patients in the control group). No grade 5 events and fatal adverse events were observed (Table 4).

**TABLE 4:** Treatment-related adverse events.

​	Treatment group n = 131		Control group n = 132	
AEs	Any grade	Grade ≥ 3	Any grade	Grade ≥ 3
Myelosuppression	62 (47.33%)	7 (5.34%)	65 (49.24%)	9 (6.82%)
Elevated ALT/AST	32 (24.43%)	3 (2.29%)	49 (37.12%)	2 (1.52%)
Elevated creatinine	3 (2.29%)	0	3 (2.27%)	0
Arrhythmia	23 (17.56%)	0	17 (12.88%)	0
Fatigue	70 (53.44%)	4 (3.05%)	74 (56.06%)	6 (4.55%)
Fever	4 (3.05%)	0	3 (2.27%)	0
Weight decreased	10 (7.63%)	0	6 (4.55%)	0
Pain	36 (27.48%)	0	42 (31.82%)	0
Anorexia	58 (44.27%)	2 (1.53%)	55 (41.67%)	1 (<1%)
Constipate	46 (35.11%)	0	40 (30.30%)	0
Hard to swallow	9 (6.87%)	0	6 (4.55%)	0
Dry mouth	44 (33.59%)	0	37 (28.03%)	0
Nausea	44 (33.59%)	1 (<1%)	37 (28.03%)	0
Vomiting	19 (14.50%)	3 (2.29%)	21 (15.91%)	5 (3.79%)
Sore throat	18 (13.74%)	0	25 (18.94%)	0
Diarrhea	18 (13.74%)	2 (1.53%)	19 (14.39%)	4 (3.03%)

Data are n (%). Adverse events of any grade occurring or grade ≥ 3 adverse events occurring in whole population (at least 1 cycle chemotherapy).

Grade 3 or greater treatment-related adverse events were myelosuppressiont (7 [5.34%] patients in the treatment group vs. 9 [6.82%] patients in the control group), elevated ALT/AST (3 [2.29%] patients in the treatment group vs. 2 [1.52%) patients in the control group), fatigue (4 [3.05%] patients in the treatment group vs. 6 [4.55%] patients in the control group), anorexia (2 [1.53%] vs. 1 [<1%]), vomiting (3 [2.29%] vs. 5 [3.79%]), and diarrhea (2 [1.53%] vs. [3.03%]).

It indicated that TCM granules did not increase the toxicity of adjuvant chemotherapy.

## 4 Discussion

The efficacy Adjuvant chemotherapy for NSCLC with a disease-free survival HR of 0.83 (95% CI, 0.74–0.94) and an overall survival HR of 0.86 (95% CI, 0.76–0.98), had been approved (Arriagada et al., 2004). Different adjuvant Chemotherapy treated with resected NSCLC showed no significant difference in overall survival, but the toxicity (Kreuter et al., 2013; Kreuter et al., 2016). The efficacy of postoperative adjuvant chemotherapy had not improved for a long time. When this clinical study was designed, chemotherapy combined with or without radiotherapy were the main standard of treatment in stage Ib-IIIa resected NSCLC. Meanwhile, First-generation EGFR-TKIs began to be reported the clinical benefits to prevent recurrence and metastasis after surgery in EGFR-mutant NSCLC. In recent years, in addition to EGFR-TKIs, Anaplastic Lymphoma Kinase (ALK)-TKIs and immune checkpoint inhibitors had been used in NSCLC patients after radical resection with ALK fusion mutation or PD-L1 TC ≥ 1%. Latest results show that, Osimertinib reduces the risk of recurrence and metastasis by 77% in NSCLC patients with EGFR-sensitive mutations after reaction and (Herbst et al., 2023), Alectinib compared to chemotherapy reduced the risk of disease recurrence and metastasis by 76% in patients with stage II-IIIa resected ALK-positive NSCLC without mature OS date (Wu et al., 2024), Atezolizumab improved a >30-month difference in median DFS compared to best supportive care in the stage II-IIIA resected NSCLC with PD-L1 TC ≥ 1% population and the OS benefit remained limited to the subgroup with TC ≥ 1% (HR, 0.47; 95% CI, 0.28–0.77),Whether in the TC 1%–49% subgroup (HR, 0.77; 95% CI, 0.56–1.06), all stage II-IIIA patients (HR, 0.94; 95% CI, 0.75–1.19), or the intention-to-treat population (HR, 0.97; 95% CI, 0.78–1.22) (Felip et al., 2025), Atezolizumab-related grade 3-4 adverse events occurred in 11% of patients (Felip et al., 2021). Compared with placebo, pembrolizumab significantly prolonged the median DFS in ITT population (53.6 months vs. 42.0 months; HR, 0.76; 95% CI, 0.63–0.91; p = 0.0014), the incidence of grade 3 or worse events was 34%, which was also higher than in placebo (O’Brien, 2022). OS data from pembrolizumab are still pending. However, patients without positive biomarkers do not get clinical benefit from these drugs, whether the benefits of DFS can translate into OS still requires more clinical evidence. Adjuvant therapy, whether using immunotherapy or targeted therapy, lasts for 2-3 years, which also faces challenges such as poor treatment adherence and the risk of adverse events from long-term medication. Therefore, many scientific questions still need to be further explored regarding current standard adjuvant therapy.

Traditional Chinese medicine is widely used in China. After more than 60 years of clinical exploration, a TCM treatment plan suitable for the Chinese population has been formed. Professor Liu Jia-xiang, the well-known master of TCM Oncologist, point out that lung cancer is a disease caused by deficiency (including yin, yang, qi, xue, et al.). After radical resection, the NSCLC patients were characterized by deficiency of righteous qi, residual toxins, phlegm and blood stasis from the perspective of TCM theory. Deficiency of righteous qi is mainly manifested in deficiency of qi, yin, both qi and yin, involving the lung, spleen, and kidney. Therefore, in the TCM treatment, strengthening the righteousness (qi, yin, qi and yin), nourishing the root, and clearing the excess toxins and suppressing pathogens are repeatedly emphasized. Differential treatment is given according to different syndromes.

A multicenter, randomised, and double-blind trial was reported to demonstrate the advantages of adjuvant chemotherapy combined with comprehensive traditional chinese medicine treatment. In NSCLC patients who were not selected for biomarker-guided therapy, oral traditional Chinese medicine and intravenous Huachansu were administered concurrently with adjuvant chemotherapy and during the consolidation phase. The results were most relevant to biomarker-negative patients or patients without access to immunotherapies.

Our research group optimised the process based on this research. After adjuvant chemotherapy (radiotherapy) combined with TCM granules was completed, four cycles of intravenous Huachansu combined with TCM granules were carried out to consolidate the effect and prevent recurrence or metastasis. Huachansu, an injectable form of Chansu, containing two primary biologically active chemical components are indole alkaloids and steroidal cardiac glycosides (Meng et al., 2012). Clinical data has indicated that huachansu may be effective in treating cancer with low toxicity and enhancing quality of life for cancer patients (Qi et al., 2011). In cancerous cells, glycosides derived from HCS exhibit cytostatic and cytotoxic activities, induce apoptosis, inhibit angiogenesis, reverse resistance to chemotherapeutic drugs, and modulate immune responses (Cheng et al., 2019). Sequential chemotherapy with Huachansu injection, combined with TCM granules, can enhance the anti-recurrence and anti-metastasis effect.

The two-year DFS rate met expectations (71.00% vs. 60.10%, p = 0.071), though the difference was not statistically significant. However, the second endpoint had showed significantly improved DFS (HR, 0.73; 95% CI 0.549–0.975) with the addition of TCM granules to standard adjuvant chemotherapy in treatment of patients with completely resected stage IB-IIIA NSCLC. We can see in the DFS survival curve graph, the two curves gradually separated by increasingly evidence since the point of approximately 4 years regardless of PP analysis or the ITT analysis (Figures 2, 3). It was also the possible reasons why the primary endpoint was not reached. This suggests that postoperative TCM treatment for NSCLC needs to be maintained for a long time, even more than 4 years. As we all know, traditional chinese medicine (TCM) treatment for malignant tumors is relatively gentle and slow-acting. By regulating the body’s physiological functions, immune system, and metabolic processes, it gradually inhibits the growth and spread of cancer cells or promotes the body’s elimination of them. This seemingly slow effect is actually long-lasting and far-reaching.

The treatment of patients following radical resection of NSCLC can result in a number of adverse effects, including surgery, chemotherapy and radiotherapy. These treatments can have a significant impact on the patient’s QOL. The administration of questionnaires regarding QOL served to reflect the symptoms and functional status experienced by patients undergoing adjuvant chemotherapy. An evaluation of the patients’ QOL was conducted at the baseline and at the conclusion of each of the two cycles, or, in the case of a single cycle treatment, at the conclusion of the cycle. The administration of TCM granules has been demonstrated to engender a significant amelioration of symptoms associated with lung cancer, including fatigue, pain, dyspnoea, shortness of breath, insomnia, cough, pain in the chest and diarrhoea. Moreover, the granules have been shown to promote physical function, emotional function and global health. In the placebo arm, symptoms of dyspnoea and insomnia also improved by the conclusion of cycle 2, as determined by both the EORTC QLQ-C30 and QLQ-LC13. Common adverse effects of surgery included shortness of breath, cough and pain in the chest, with these effects being gradually reversible post-surgery. The combination of adjuvant chemotherapy and TCM granules has been shown to facilitate a more rapid recovery from cough.

In the present study, the safety profile of the treatment was found to be consistent with the findings of previous reports in the field of chemotherapy. The most prevalent grade 3 or higher treatment-related adverse events were hematological toxicities, liver damage, fatigue, and vomiting. The investigation revealed no significant increase in adverse events in conjunction with the incorporation of TCM granules. The gentle nature of TCM reduces side effects and complications during treatment.

This study still had many limitations: (1) It was non-significant in primary endpoint (2-year DFS p = 0.071) despite potential median DFS benefit, it would be needed further verification; (2) Because 30% of the patients in this study were lost to follow-up after disease progression, overall survival data were unavailable despite a median follow-up of 90.2 months; (3) A total of 23 patients were excluded. 11 patients presented with early recurrence or metastasis (<4 months), attributed to either surgical failure or metastasis. Furthermore, 12 patients withdrew from the prescribed protocol (TCM granules and intravenous preparations) to other treatment, rendering their data unsuitable for analysis; (4) Postoperative radiotherapy was administered to patients with >3 positive N2 stations; (5) Inclusion required specific TCM syndrome differentiation. We focused on the three primary lung cancer syndromes, which account for most patients after radical resection of lung cancer; (6) Patients were enrolled between 2016 and 2018, a period before targeted and immunotherapies were approved for postoperative NSCLC patients. Therefore, the results of this study are more applicable to postoperative NSCLC patients who did not receive targeted or immunotherapy; (7) Our study conducted a clinical observation of the efficacy of comprehensive TCM treatment. Further research on the specific mechanisms of action may require a combination of methods such as network pharmacology, chromatographic analysis, and metabolomics, which is one of the directions of our future research.

In the future, based on the subgroup analysis of the results of this study, we will further design a randomized controlled multicenter study with DFS as the endpoint to further observe the exact effect of traditional Chinese medicine on prolonging DFS. We can also design corresponding animal and cell experiments to elucidate the molecular mechanisms by which TCM prevents recurrence and metastasis. Furthermore, TCM treatment for individuals with biomarker-negative patients, or patients who had not received immunotherapy may fill the gap following adjuvant chemotherapy.

## 5 Conclusion

In summary, this multicentre, double-blind, randomised clinical trial of radical resection NSCLC demonstrated that the addition of traditional Chinese medicine granules to platinum-based chemotherapy and sequential huachansu injection as adjuvant treatment tended to improve 2-year DFS and had the potential to extend the DFS function. TCM granules can significantly relieve lung cancer-related symptoms such as fatigue, pain, dyspnoea, breathlessness, insomnia, coughing, chest pain and diarrhoea, and promote physical and emotional function and overall health. There was no evident increase in adverse events associated with the addition of comprehensive traditional Chinese medicine treatment.

## Data Availability

The raw data supporting the conclusions of this article will be made available by the authors, without undue reservation.
